# The infant mummy’s face—Paleoradiological investigation and comparison between facial reconstruction and mummy portrait of a Roman-period Egyptian child

**DOI:** 10.1371/journal.pone.0238427

**Published:** 2020-09-16

**Authors:** Andreas G. Nerlich, Lukas Fischer, Stephanie Panzer, Roxane Bicker, Thomas Helmberger, Sylvia Schoske

**Affiliations:** 1 Institute of Pathology, Academic Clinic Munich-Bogenhausen, München, Germany; 2 3-D-Construct, Köln, Germany; 3 Department of Radiology, Trauma Center Murnau, Murnau, Germany; 4 Department of Radiology, Paracelsus Medizinische Universität, Salzburg, Austria; 5 Staatliches Museum Ägyptischer Kunst München, München, Germany; 6 Institute of Diagnostic and Interventional Radiology, Academic Clinic Munich-Bogenhausen, München, Germany; University of Florence, ITALY

## Abstract

In Graeco-Roman times in the Lower-Egyptian Fayoum region, a painted portrait was traditionally placed over the face of a deceased individual. These mummy portraits show considerable inter-individual diversity. This suggests that those portraits were created separately for each individual. In the present study, we investigated a completely wrapped young infant mummy with a typical mummy portrait by whole body CT analysis. This was used to obtain physical information on the infant and provided the basis for a virtual face reconstruction in order to compare it to the mummy portrait. We identified the mummy as a 3–4 years old male infant that had been prepared according to the typical ancient Egyptian mummification rites. It most probably suffered from a right-sided pulmonary infection which may also be the cause of death. The reconstructed face showed considerable similarities to the portrait, confirming the portrait’s specificity to this individual. However, there are some differences between portrait and face. The portrait seems to show a slightly older individual which may be due to artistic conventions of that period.

## Introduction

A custom specific to the Ancient Egyptian Graeco-Roman period, found in numerous burials of that period, was the placement of mummy portraits over the embalmed face, while the rest of the body was wrapped in linen bandages according to the typical ancient Egyptian funerary rituals [1]. Since they were first mentioned by the Austrian art dealer Theodor Graf in 1887, approximately 1,000 such portraits have been discovered [2], most of which have been found in the Fayoum area. Currently, several hundreds of those portraits are assumed to exist in various museums and collections, either as isolated objects or as parts of complete mummies. However, only as little as an estimated 10% of the portraits are still attached to the mummy [2, 3].

Those mummy portraits seem to go back to the Roman customs of funerary portraits that were assumed to represent the deceased for the afterlife. In the Imperial Roman mainland, often funerary stelae were made of stone which were placed near the deceased´s urn; however, as shown e.g. in the case of Julius Cesar, also wax effigies were used as memories providing very realistic representations of the individual (also called the “Republican Roman portraiture”) [4]. This custom may have influenced the Egyptian part of the Roman Empire, where they were manufactured as paintings on small boards of very durable wood, painted with either coloured beeswax (called encaustic) or tempera colours [1, 5].

A few previous studies have dealt with the probability that the portrait might indeed represent the buried individual´s face [2, 6]. Likewise, recent studies have collected comparisons of published facial reconstructions of such burials to their portraits, coming to very divergent results ranging between complete concordance to absolute divergence [5, 6]. All these investigations have been done on adult mummies, none on a sub-adult individual.

In the present study, we provide the first scientific report of a facial reconstruction of an infantile Ancient Egyptian mummy from the Roman period that has been compared with its mummy portrait. Beyond the mere reconstruction of the facial traits, we have investigated the degree of concordance/divergence, the possible reasons therefore and the more general implications for the interpretation of mummy portraits. Furthermore, the extensive radiological investigation of the infant mummy gives insight about the physical properties and diseases in infants of the period.

## Material and methods

### The infant mummy

**Fig 1:**
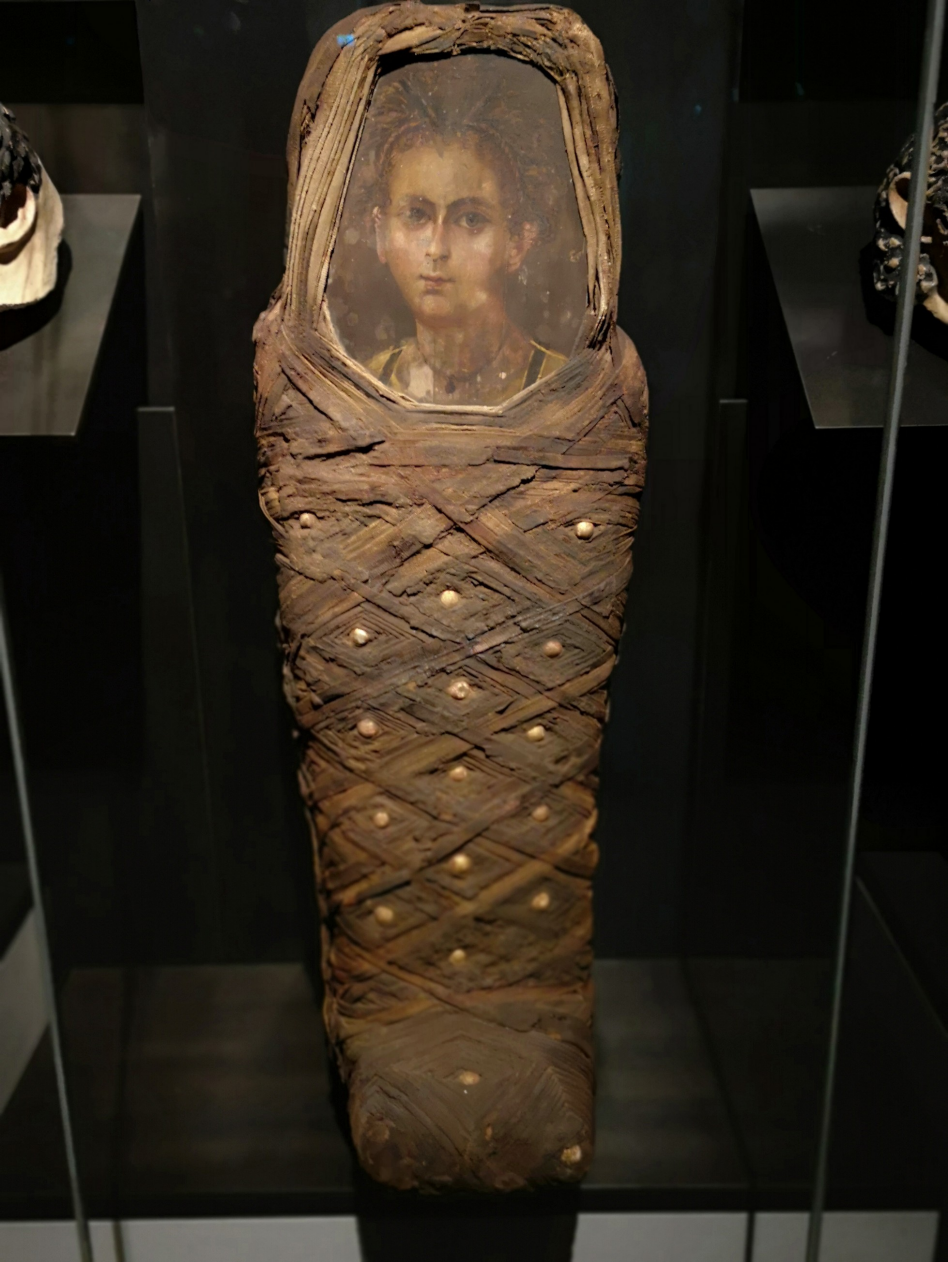
The mummy ÄS 1307.

The mummy (Fig 1) is part of the collection of the Staatliches Museum Ägyptischer Kunst (SMAEK) München (Gabelsbergerstrasse 35, D-80333 München; www.smaek.de) where it is registered under the number ÄS 1307. It has been housed there since 1912, when the famous Egyptologist Sir Flinders Petrie donated the mummy to the Royal Bavarian Collection of Antiquities. The records of this object indicate that Sir Petrie had discovered the mummy in the 1880s in a cemetery close to the pyramid of Hawara in Lower Egypt, near the Fayum region, which is well known to have harboured numerous Roman period settlements with related cemeteries [7].

The mummy is 78 cm long and 26 cm wide. In addition to the portrait, the body is completely covered by layers of criss-crossed linen bindings with several symmetrically arranged gold-plated plaster buttons. The feet are placed in a standing position. The wrapping has obviously been carried out very carefully, in a cassette pattern.

**Fig 2:**
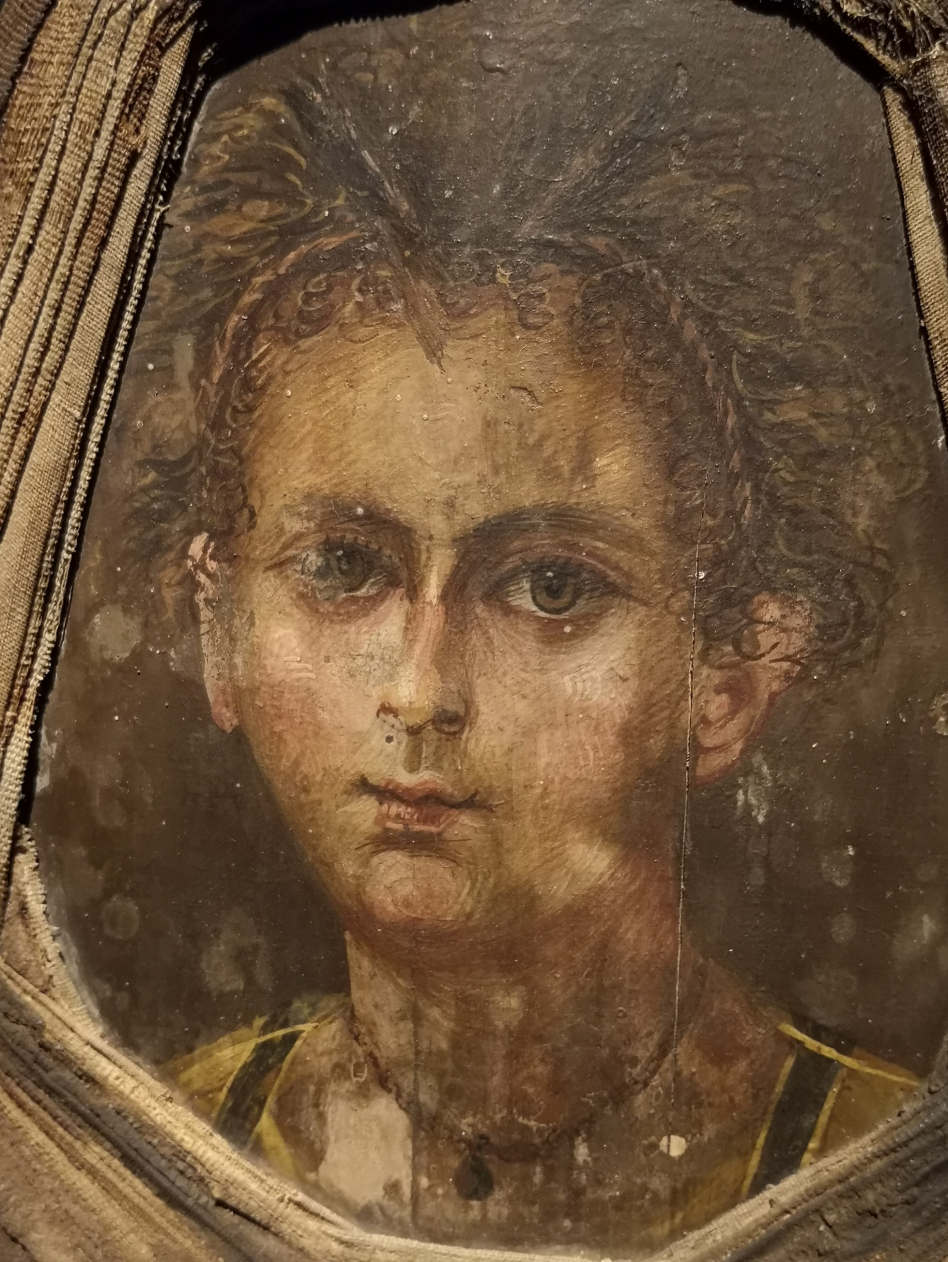
The encaustic mummy portrait.

The mummy portrait shows an infant with curled hair woven into two hair strands running from the crest to the ears. The individual has large eyes of brown colour, a long, thin nose and a small mouth with full lips. A necklace with a small medallion hangs around its neck (Fig 2). The skin colour was evaluated using the dermatological skin colour scale by Fitzpatrick [8]. This grading system describes the human skin colour in 6 types (types I–VI). Within this system, the portrait belongs to a type IV skin colour.

### Previous radiological examination

The mummy had already been investigated by x-rays in 1984 [7]. The complete body had been radiographed and the images then merged into a whole-body image. The original x-rays were available for the present study.

### CT-examination

**Fig 3:**
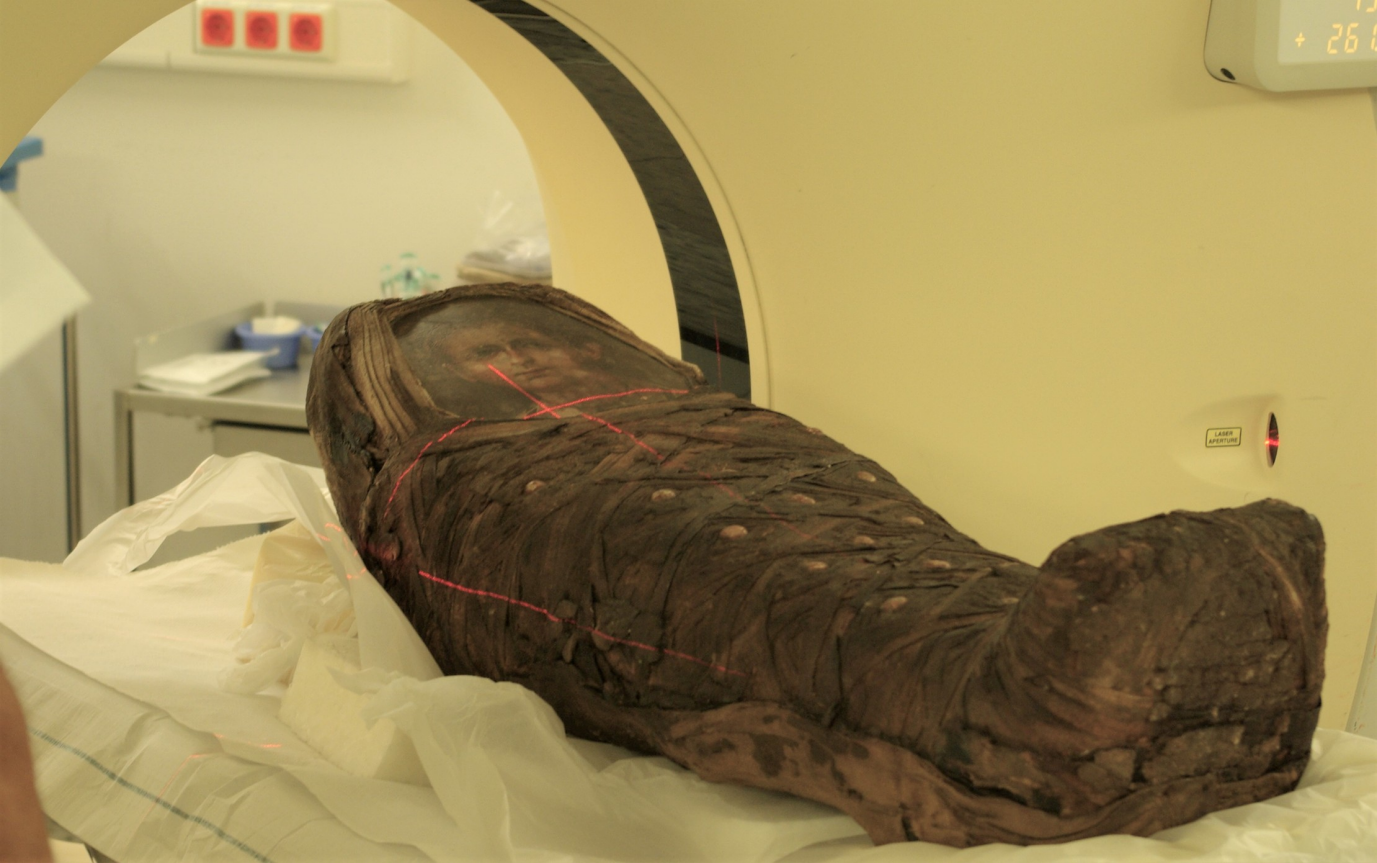
The infant mummy during the CT-analysis.

In order to find out more about the individual, the mummy was thoroughly investigated using a full-body CT scan (64-row detector CT, LightSpeed VCT, General Electrics, Milwaukee, Wisconsin, USA) in supine position (Fig 3) with a slice thickness of 0.625 mm, interval of 0.625 mm, 120 kV and 200 mA in a standard algorithm, as previously done [9]. Additional three-dimensional and multi-planar reconstructions as well as maximum intensity projections were prepared on the corresponding workstation (ADW 4.4, General Electrics, Milwaukee, Wisconsin, USA).

### Facial reconstruction (see also supplementary material)

The facial reconstruction was prepared using the CT-data set that had been obtained for the skull [9]. The entire virtual reconstruction was produced with the 3D software Blender; this open-source 3D and animation software was applied in the same way as in previous studies [10].

In a first step, the skull was virtually orientated into a neutral position given by a putative line between the lower margins of the orbitae and the porion (so-called “Frankfurter Horizontale” = Frankfurt plane). Subsequently the eyes were rendered based on a mean eyeball diameter of 22 mm, adjusted to take into account the age of the individual. The eyes were oriented along the tangent between the supra- and infraorbital line; the eyeball was placed slightly below the line between the fronto-maxillary suture and the zygomatico-frontal suture. The nose was reconstructed according to the Lebedinskaya method, where the piriform aperture is mirrored externally.

The facial form is predominantly determined by the soft tissue. The thickness of this soft tissue layer was reconstructed according to the data given by Manhein et al. [11], which has established reference values for infantile facial soft tissue for Caucasians between 3 and 8 years of age. This data was collected by means of ultrasound scans on living individuals. Beyond the data for the face, the residual skull data, including the crest, were defined according to the skull bone morphology. In the mandibular region, a mean soft tissue cover was assumed in line with early infantile face profiles. Here, slightly interpretative adjustments were added to the raw data to include presumed slightly higher fat pads in early infantile age. The width of the orifice was determined by the position of the canine teeth [12], which also determines the shape and width of the lips.

In a next step we applied skin colour in accordance with the colour determination by the dermatological scale as indicated above. Besides the colour, the 3D skin material is also made of multiple textures defining the reflections and the fine details. On the lips, nose and ears, a slight translucency effect (subsurface scattering) was used.

In order to avoid bias, the facial reconstruction artist was carefully kept away from any images or specific information concerning the portrait. Only at the aforementioned last stage of reconstruction were the colour of the eyes and hair as well the type of hairstyle communicated to the artist in order to assure that the data for these three features would match. Hairstyle and eye colour were manually corrected to conform with the information received.

With the finalization of the facial reconstruction came the first full exchange of data for the reconstruction and the mummy portrait. For further evaluation, the virtual image was compared to biometric data obtained from the portrait. Accordingly, certain specific proportions and dimensions were compared, such as the distance between the horizontal eye line, mouth and nose, but also the width of the eyes, nasal bridge and mouth opening.

## Results

### Whole-body x-ray of the mummy

**Fig 4:**
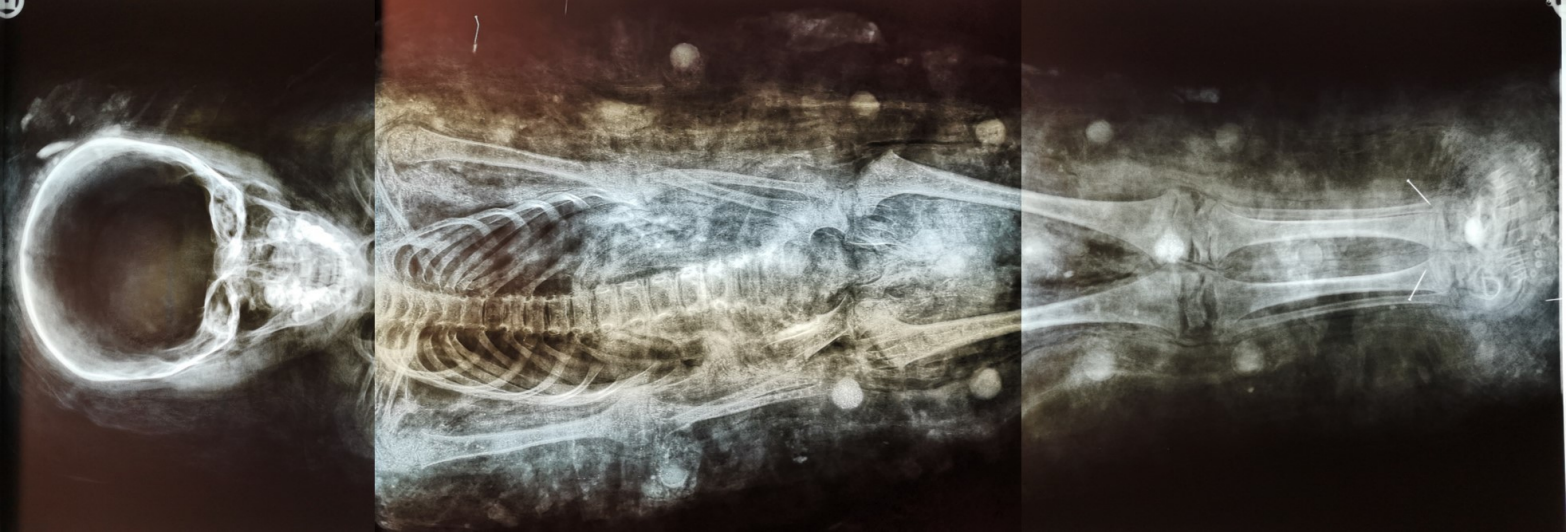
Whole body x-ray of the infant mummy.

The complete body radiograph of the infant that had been prepared during the first examination of the mummy in 1984 (Fig 4) shows a complete infantile human body in correct anatomical position. According to the long bone size and the dentition (as far as was detectable on the antero-posterior positioning of the body), the individual’s age was determined to be 4–6 years. Several skeletal elements were slightly displaced–most obviously due to post-mortal shrinkage of the body during dehydration. No further biographic or pathological data could be ascertained; in particular no evidence was obtained for the individual’s sex. Furthermore, no foreign bodies (such as amulets) could be seen just as no soft tissue structures could be evaluated [7].

### CT-morphological observations–signs of funerary and post-mortem manipulations

**Fig 5:**
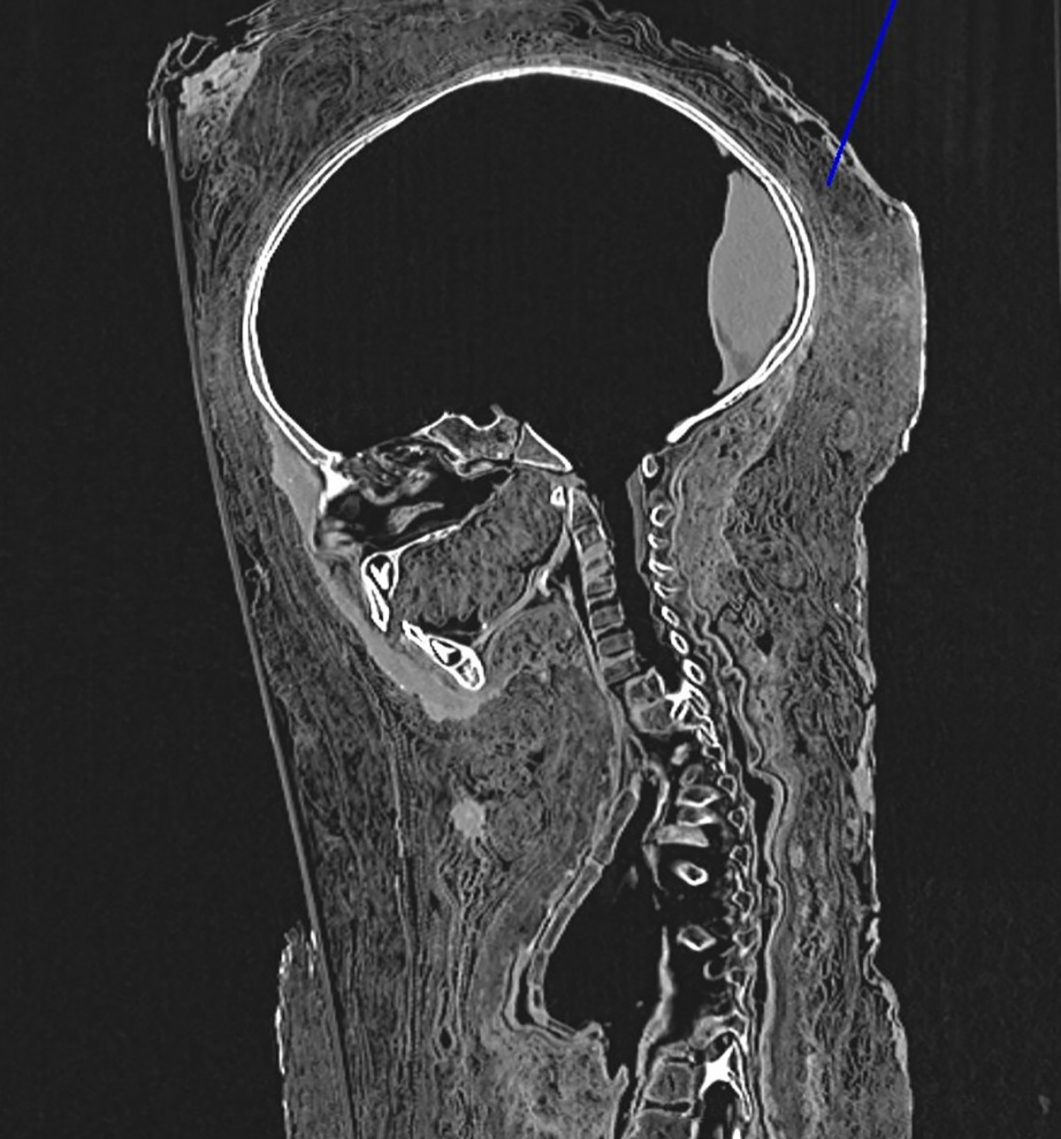
CT-scan section through skull and thorax. This shows the vertical plane of the embalming substance at the occipital skull cavity, the ethmoidal osseous defect and the linen filling of the mouth.

During the present CT-scan analysis, the skull was found to be empty except for the dorsal skull fovea where a 6.5 x 8.5 x 2.6 cm large amorphous foreign material mass of presumably resinous origin–forming the typical vertical reflection caused by embalming substances within the skull–is visible (Fig 5). This material was obviously introduced into the emptied skull through a transethmoideal defect to the left ethmoid region, resulting in the typical destruction of the left lamina cribrosa (Fig 5). The left nasal cavity is deviated to the right side, the lower nasal concha and the right middle concha are preserved; the other structures are missing. Instead, the left nasal cavity is filled by a piece of linen reaching up to the ventral skull base. The mouth is similarly filled with linen tissue.

**Fig 6:**
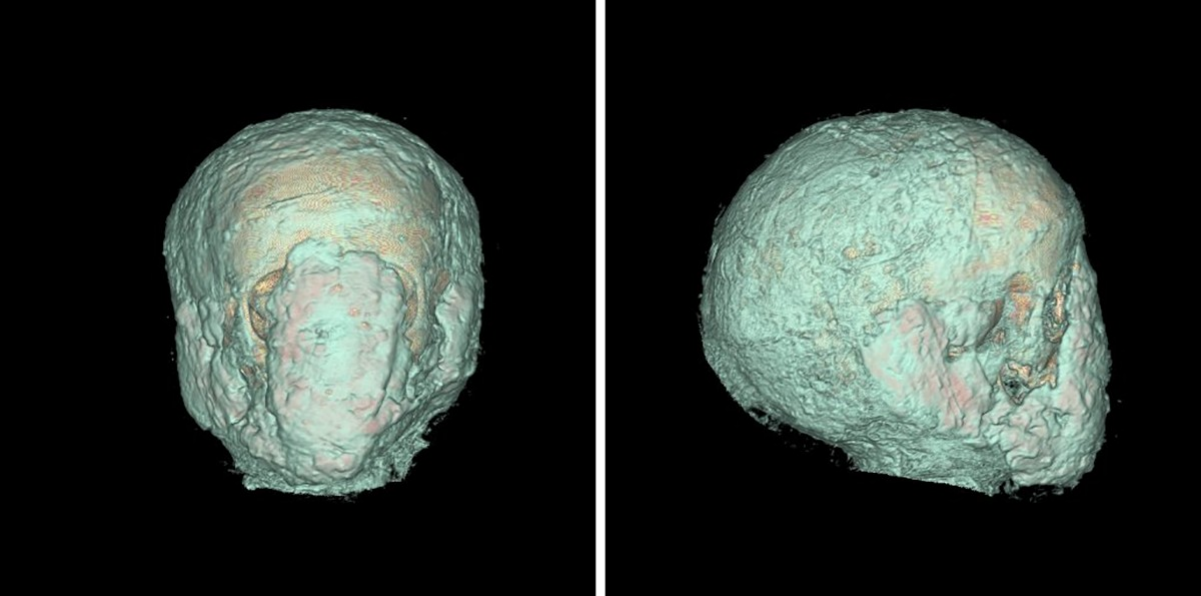
Three-dimensional reconstruction of the CT scans. This shows an amorphous material mass over the face–obviously for the proper fixation of the portrait; left side: frontal view; right side: lateral view.

The face and both ears are covered by a homogeneous and amorphous foreign material mass that obviously forms a basis for the proper fixation of the mummy portrait (Fig 6).

**Fig 7:**
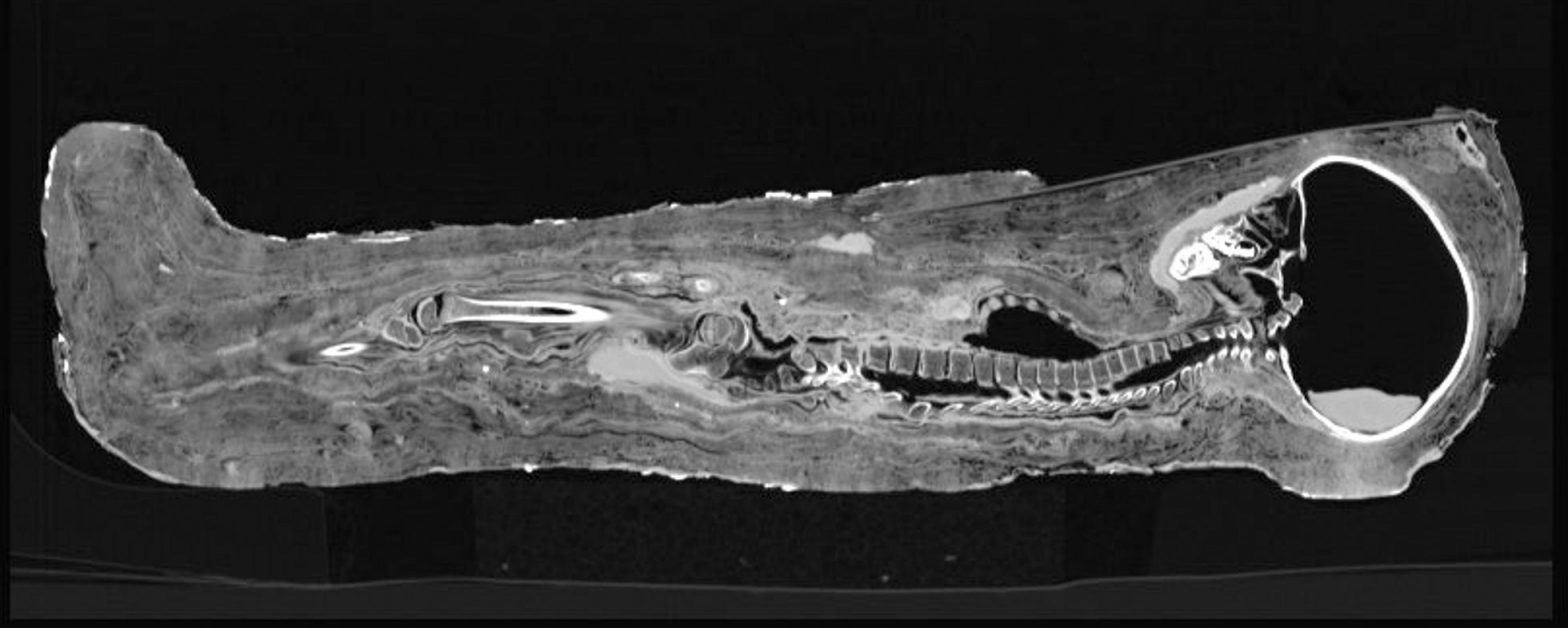
Longitudinal section through the complete mummy. Over the face, the mass is well identifiable, the body is otherwise tightly wrapped and there is no shrinkage of the body below the bindings.

Otherwise the body is tightly wrapped in numerous layers of linen bindings, with the innermost being almost attached to the body surface so that no major post-mortal shrinkage of the cadaver can have been occurred (Fig 7).

The rectal canal is filled with foreign material which is in connection to a material mass filling out the space between the lower margins of the pelvis and the proximal femora on both sides and extending to the adjacent dorsal zone.

### Radiological estimation of age and sex

The individual’s sex was easily determined by the presence of a very well-preserved external male genitalia.

The individual´s age was estimated using body and bone length metrics, bone ossification, tooth development, and supplementary observations.

The body length of 78 cm and long bone measurements indicate an individual (infantile male) aged 3 years +/- 12 months. Bone ossification–in particular that of the carpal bones–is most consistent with 3–4 years of age. Dental age pattern with apparent deciduous teeth D1-5 in upper and lower yaws is in favour of an individual of 3–5 years of age. Frontal and sphenoideal sinuses are still not pneumatized, the mastoids are only slightly pneumatized. The apex of the second vertebral body (*dens axis*) is not yet ossified, the dens and the corpus are still separated. In summary, most indicators suggest an age between ca. 3–4 years.

### Norm variants and post-mortem alterations

The skull is elongated and shows a prominent *Os occipitale*. In addition, the dorsal arch of the first vertebral body (*atlas*) is non-ossified, its margins appear sclerotic so that an inborn anomaly–without clinical relevance–is suggested. A small additional rib is visible in the position of the 7^th^ cervical vertebra on both sides. The rest of the vertebral column is slightly distorted and shows several vertebral bodies in luxation position–most presumably as the results of post-mortem shrinkage. The coccygeal bone is non-ossified and slightly dislocated dorsally.

In addition, a duplication of the bone nucleus on both proximal humeri is evident, the right clavicle is slightly deformed and the thorax is asymmetrically arranged, the latter possibly also being the consequence of post-mortem shrinkage. The xiphoid process is inverted (*Pectus carinatus*). Dislocations of the ventral pelvic ring and several major joints are obviously also the result of post-mortal dehydration effects.

**Fig 8:**
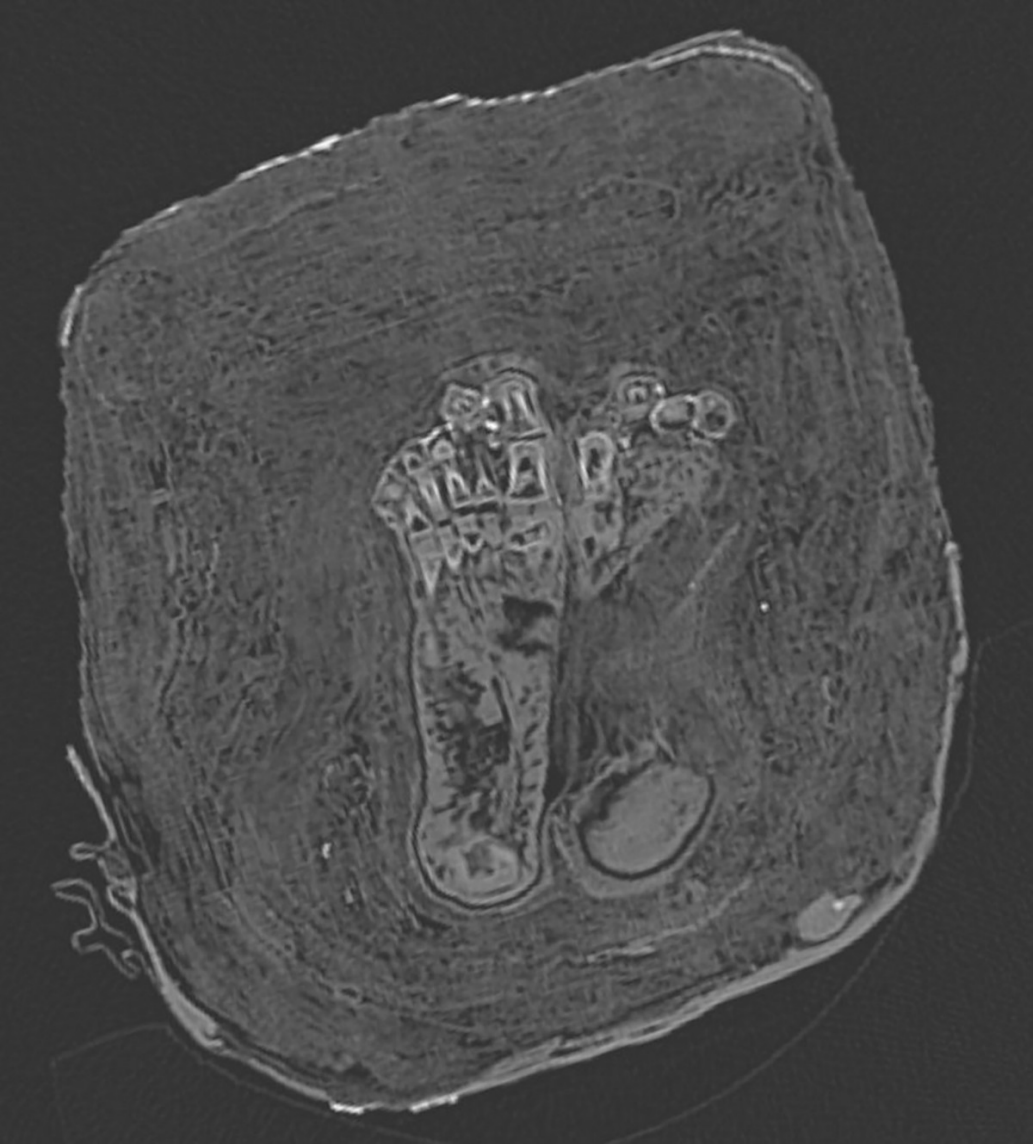
Reconstruction of the feet. This shows a so-called “Greek foot” variant, i.e. the big toe is shorter than the second toe.

Finally, the infant has a so-called “Greek foot”, i.e. the second toe is longer than the big toe on both feet (Fig 8).

### Internal organs and pathological findings

In the central mediastinum, residues of the heart are visible. These are unremarkable, as are remnants of the liver (9 cm in length).

**Fig 9:**
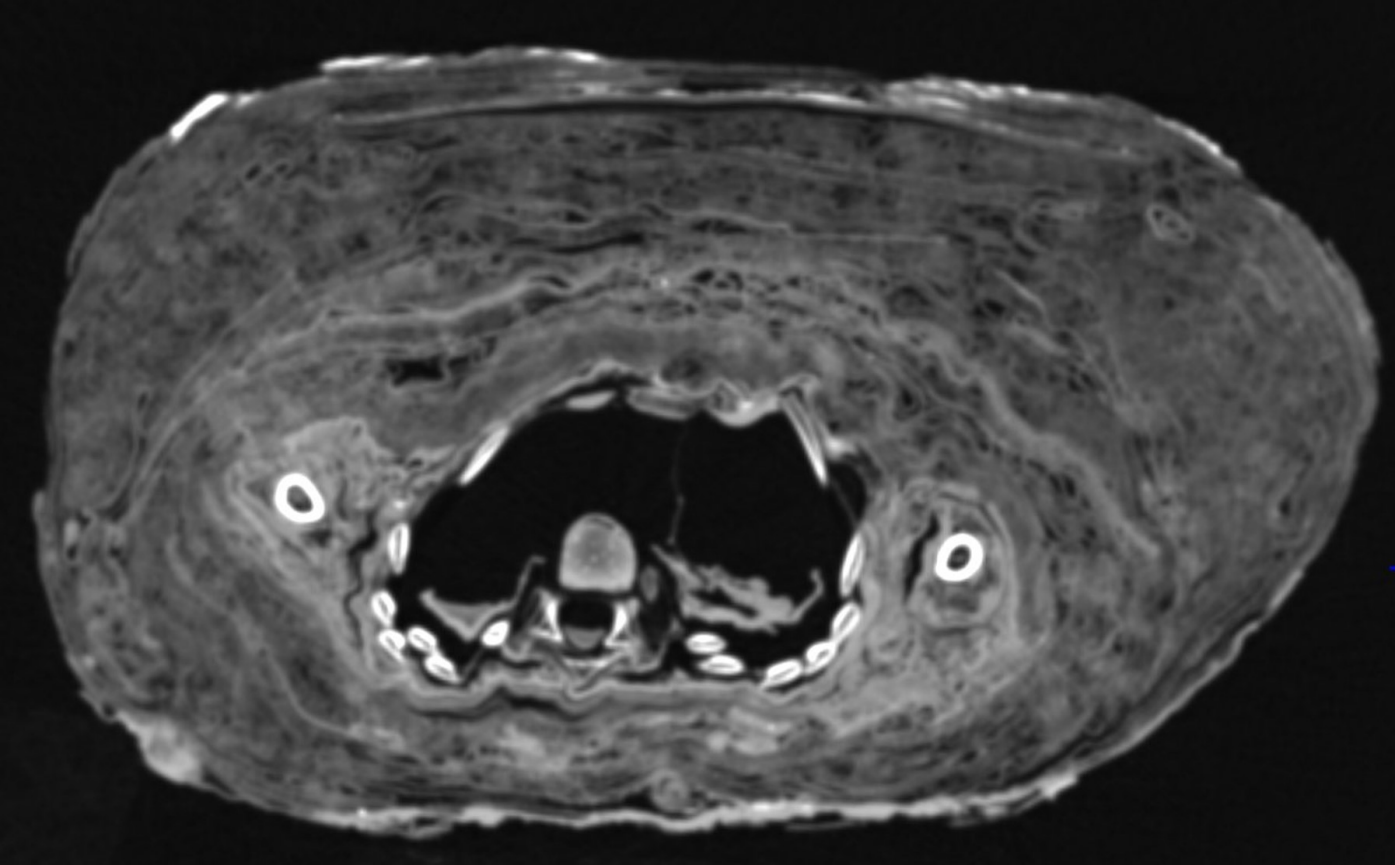
CT-scan through the chest. This section shows the residues of both lungs; the right lung being patchy condensed.

Most remarkably, however, the lungs bilaterally show an irregular patchy condensation of the parenchyma highly suggestive of the residues of pathologic infiltrations, such as in pneumonic inflammation. In accordance with these findings, the right pleural cave reveals a condensed layer that could be the residue of a pathologic exudation (Fig 9).

### Result of the facial reconstruction

**Fig 10:**
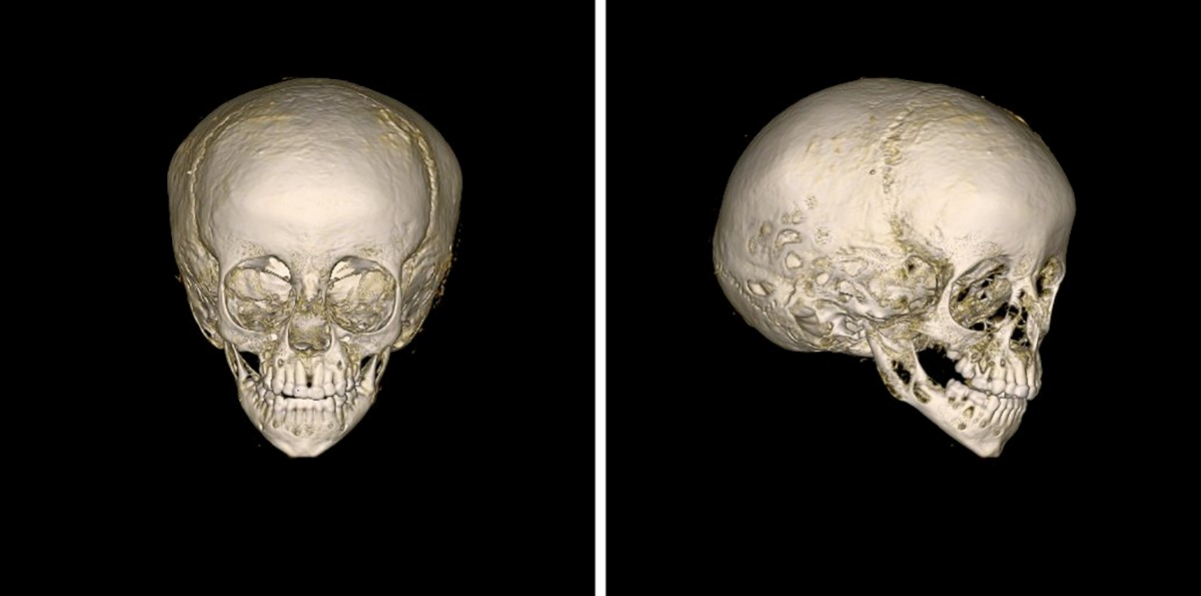
Three-dimensional reconstruction of the skull. On the left side: frontal view; on the right side: frontolateral view.

The CT data for the skull (Fig 10) allowed the virtual reconstruction of the face. The use of reference values for the soft tissue and the placements of the eyeballs, shape and size of the nose, insertion of the ears etc. were done according to available standard data, with some minor artistical corrections. The colour of skin and hair, as well as the form of the hairstyle were adjusted at the end of the process to correspond to the information given by the portrait (see above).

**Fig 11:**
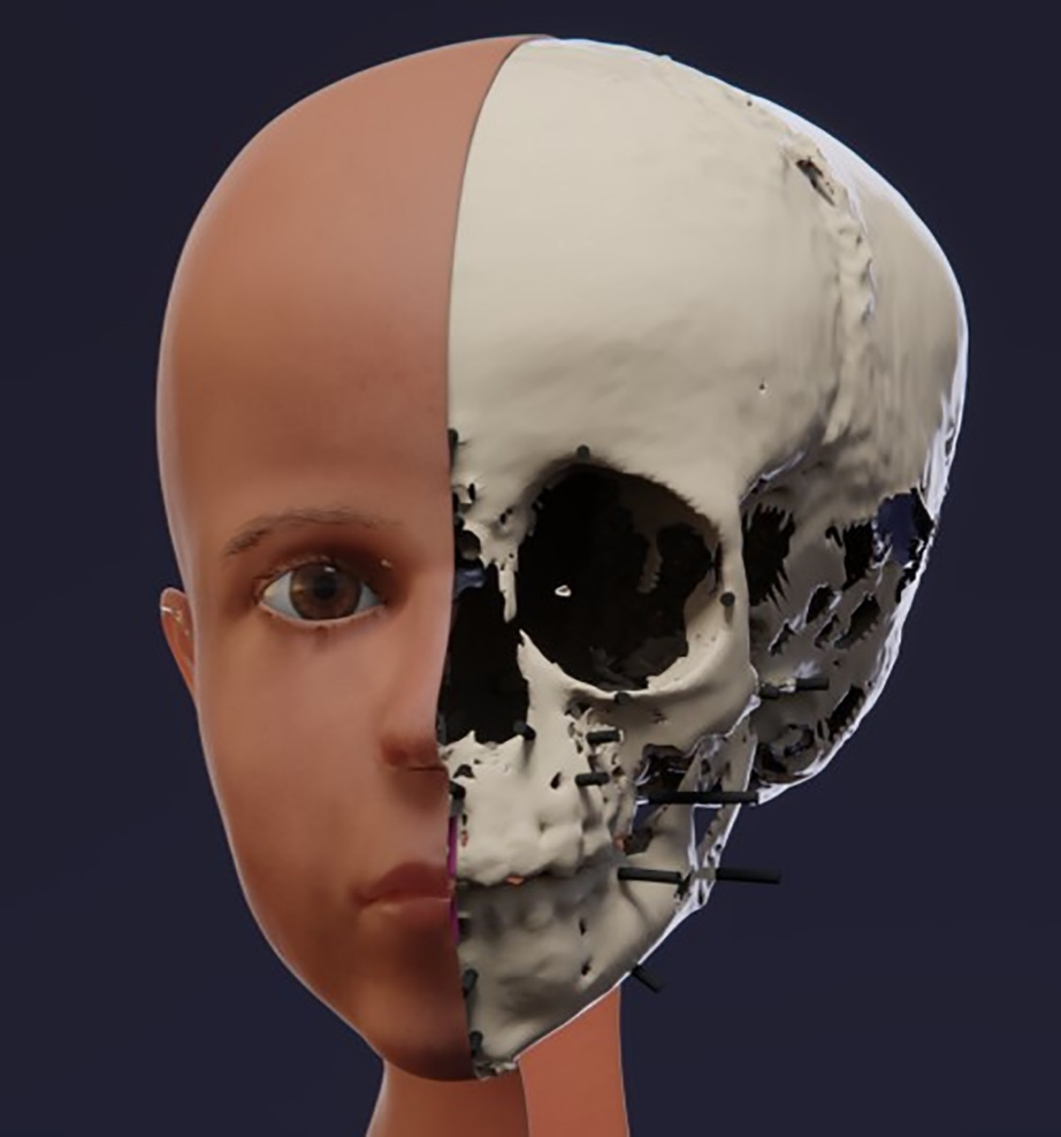
Facial reconstruction showing the positioning of the soft tissue upon the skull.

**Fig 12:**
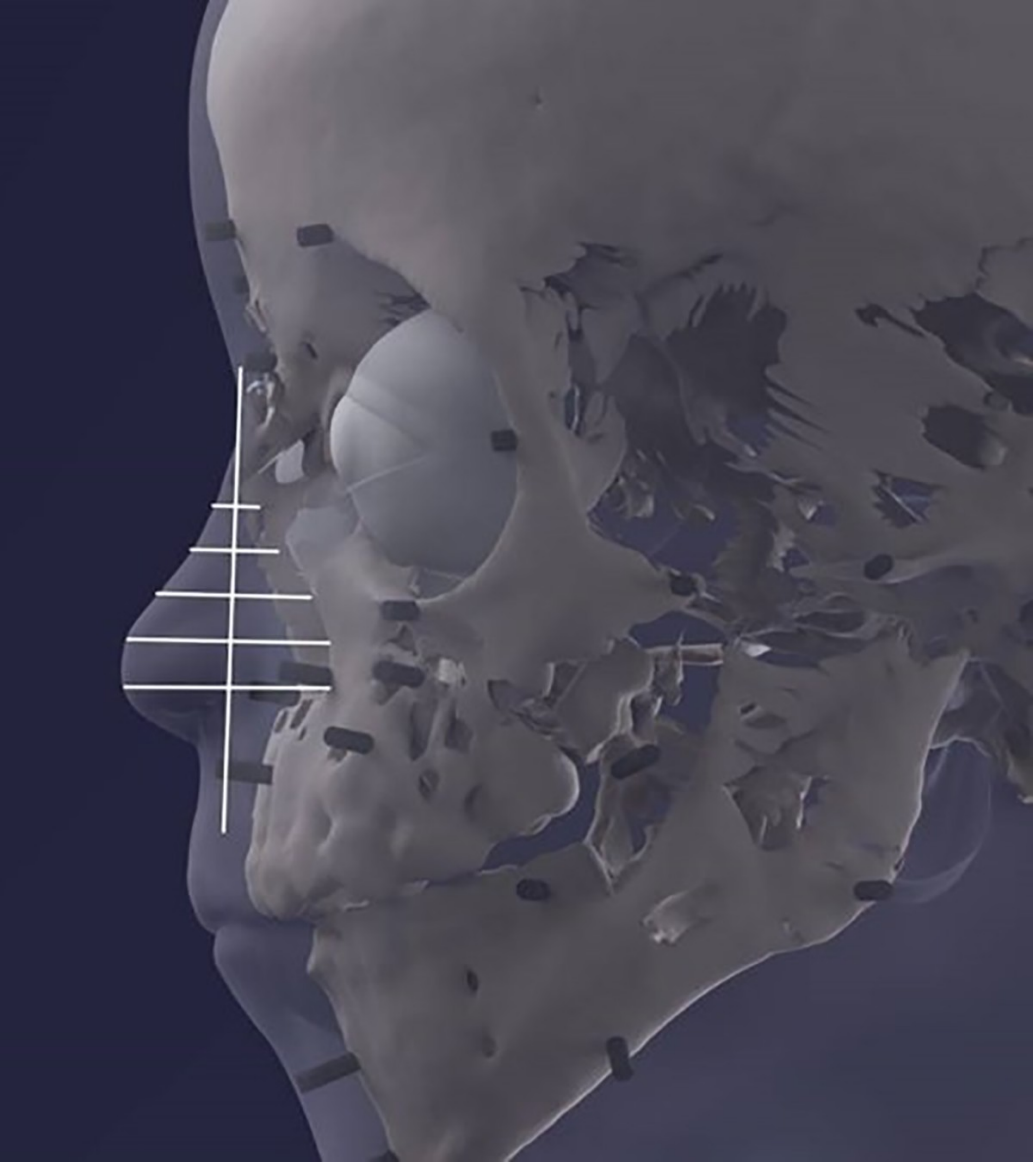
Reconstruction of the nose.

**Fig 13:**
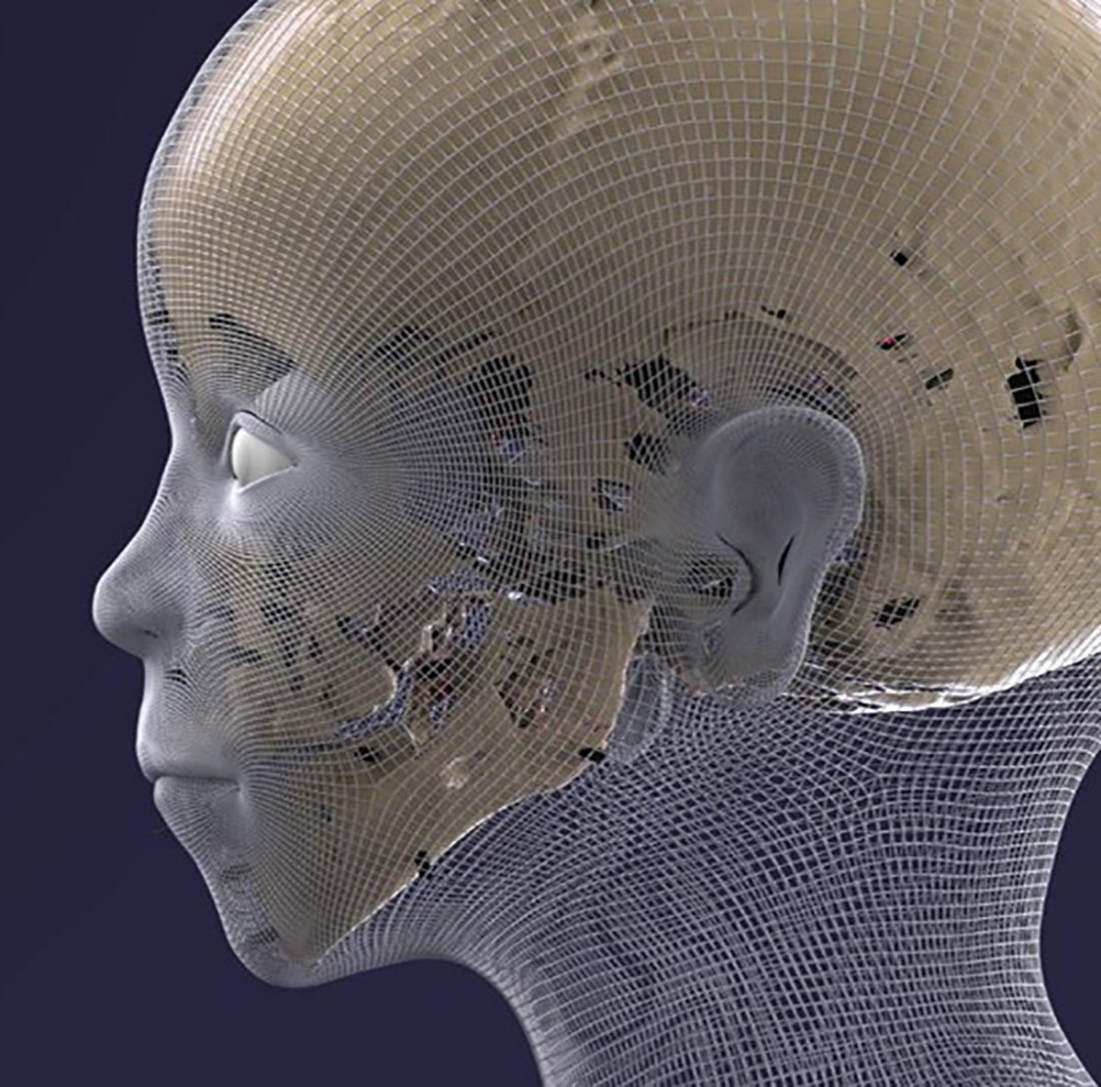
The mesh surface following the application of the virtual soft tissue distance holders.

**Fig 14:**
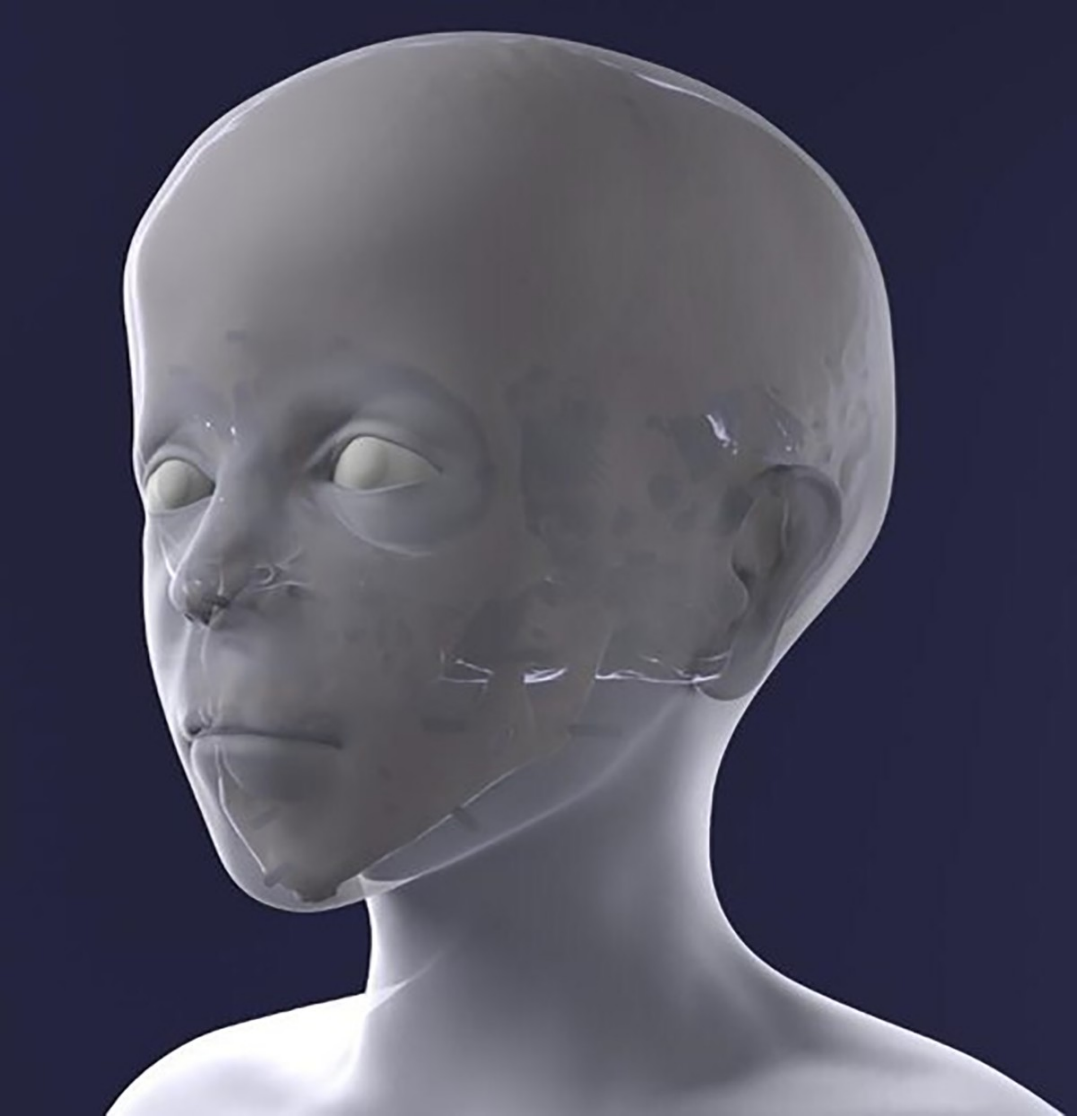
The remodelled face without coloration of surface and eyes.

**Fig 15:**
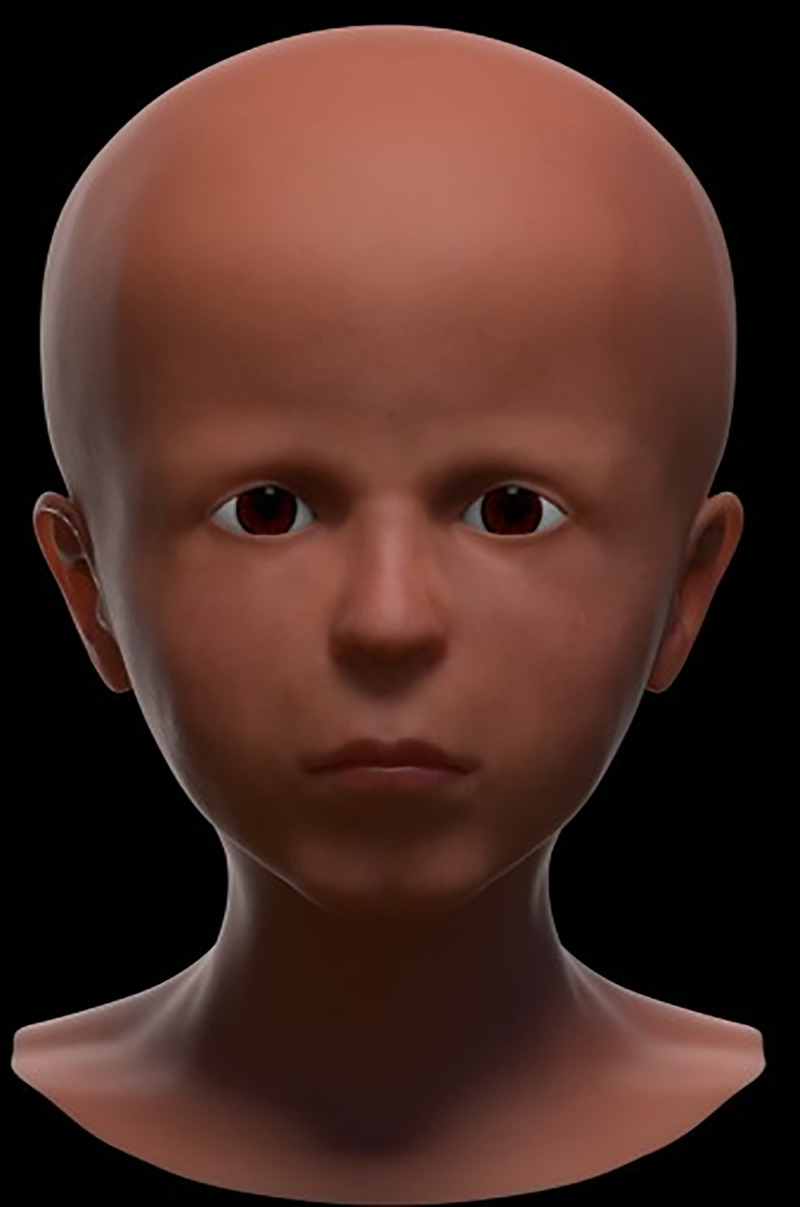
Final “raw model” of the reconstructed face following coloration of the surface.

**Fig 16:**
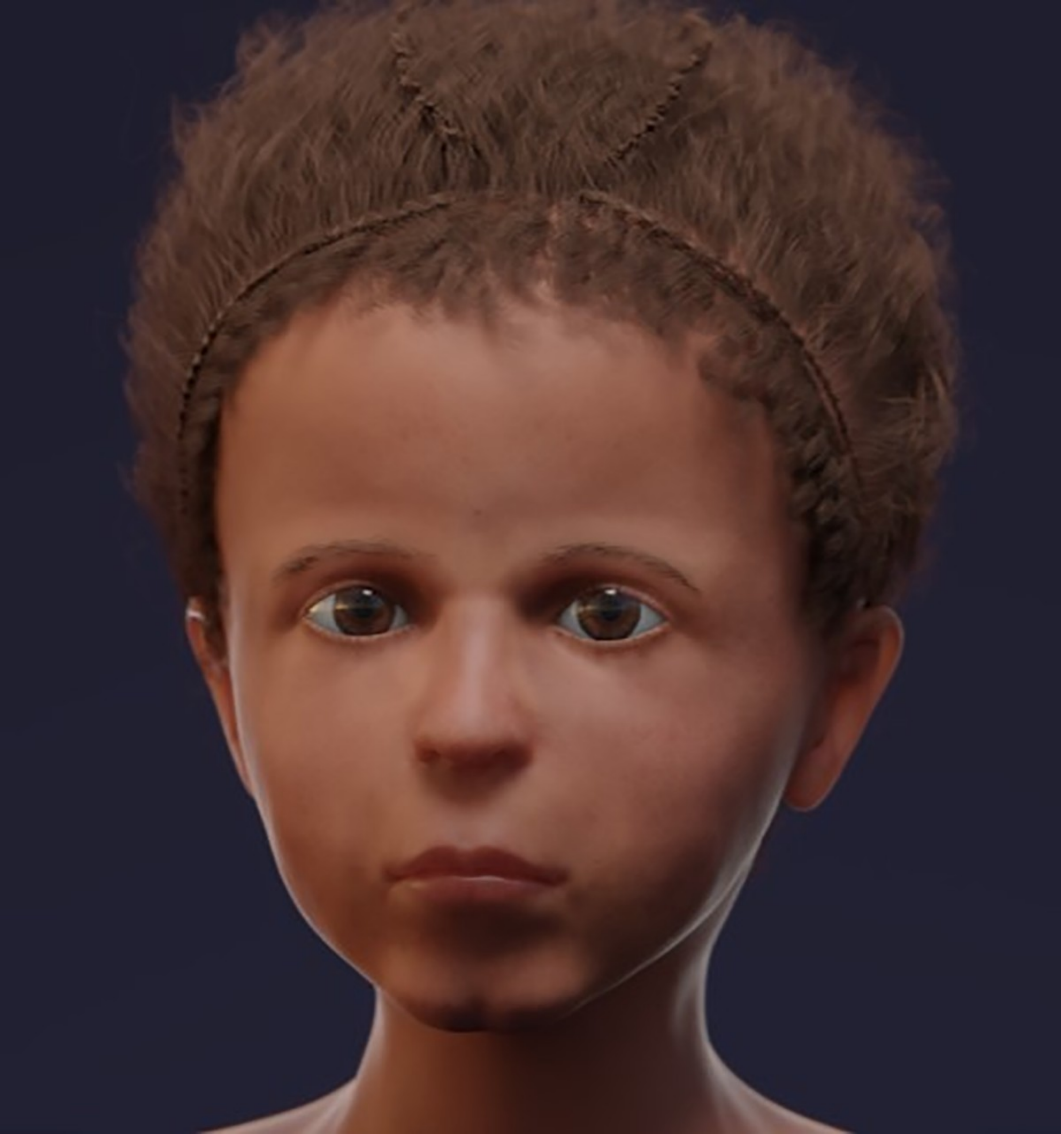
The final version of the infant’s reconstructed face, including the hairstyle.

The reconstruction process provided step-by-step data on the face, which are shown in Fig 11–16 (additional information is presented in the supplemental material/ videos).

**Fig 17:**
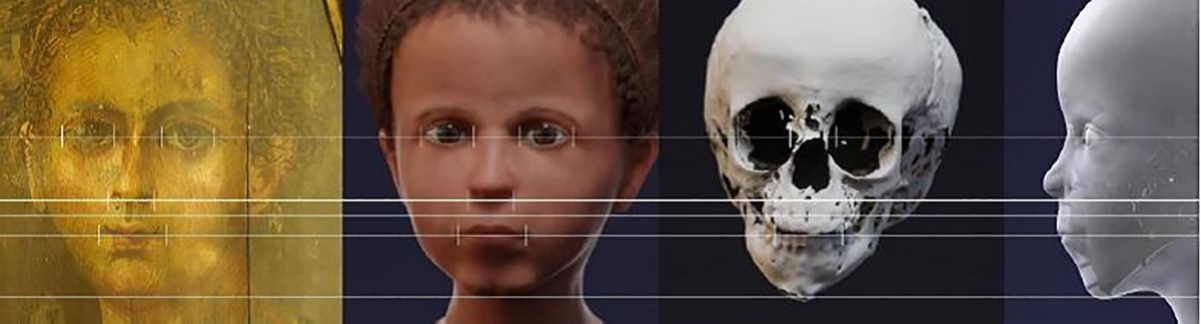
Biometric comparison of the portrait and the reconstructed face, also taking the skull into account.

The facial reconstruction shows a child with typical infantile facial features very similar to those of the portrait. On the biometrical level, the proportions between the dimension of the forehead to the eye line, the distance to the lower nasal aperture and the mouth opening were exactly the same between portrait and reconstruction. However, differences existed between the width of the nasal bridge and the size of the mouth opening with both being more slender and “narrow” in the portrait than the virtual reconstruction (Fig 17).

A comparison between both images on a subjective (sentimental) visual basis performed by 10 individuals professionally engaged in “image analysis” (mainly routine anatomic and surgical pathologists from our institution) revealed a subjective concordance between both ranging between 30 and 90% with a mean value of 66,6% on a 0–100% scale (none to absolute concordance). Interestingly, the evaluation by four female judges were much more closely grouped (50–70%) than those by six male evaluators (30–90%) suggesting slight intergender differences in the subjective evaluation of a face.

## Discussion

Since their first descriptions in 1877 by the Austrian art dealer Theodor Graf, the so-called “mummy portraits” have been attributed to the funerary practices of a particular group of Ancient Egyptians in the Graeco-Roman period [1]. The portraits show male, female, juvenile and infantile faces of all age classes (except newborns) with very high inter-individual diversity of facial features but a constantly similar pattern of presentation, with the head in nearly frontal position and ending at the upper chest [1, 2]. Every portrait also has individual features beyond the mere face, such as different hairstyles, jewellery and details of their clothing.

Despite this circumstantially high degree of individualism in the portraits, it has been a constant matter of debate whether those portraits really represent the deceased or not. The as yet most extensive study on this subject by Brier and Wilkinson [2] collected 5 cases with a comparison between the portrait and a facial reconstruction that had been made upon the mummy skull, which was visible due to damage to the mummy bandages. These few cases were reconstructed using mostly a modified method developed by Gerassimow in the 1930s. Both the original and the modified method (developed by Wilkinson and Neave) require a “physical” skull replica which is used for the application of soft tissue layers according to established values. The replicas in the study had been made either by direct duplication of the osseous skull or through 3D-printing for skulls that had been previously evaluated by CT scanning. In addition to this collection, isolated further reconstructions are available in literature on Egyptian mummies [13–16] but also on other historic individuals [17, 18].

These previous papers raised the following principle questions [2]: “When were the portraits painted? Were they done from life and only later bound into the wrappings of the mummy? Or were they done posthumously? And–probably most important–do they really portray the deceased or are they stylized?”

Since most of the portraits studied come very close to the reconstructed face, is has been postulated that the portraits really represent the deceased. Brier and Wilkinson [2] further suggest that they had not been manufactured posthumously, but prepared during the individual’s lifetime and kept at home. The strongest argument for this interpretation comes from an observation by Sir Flinders Petrie who had detected that virtually all portraits had been cut down from their original size to fit within the final mummy bandages. Furthermore, Petrie found one portrait within a tomb that had not (yet) been affixed to the mummy.

In contrast, there are several examples where the reconstructed face and the portrait are extremely divergent. Likewise, one of the portraits shows a young man while the mummy is that of an elderly man with a white beard [1]. In addition, Brier and Wilkinson [2] report one case (“The Glyptothek Mummy”) where the reconstruction and portrait show individuals with completely different ancestries.

While it might be conceivable that adult individuals stored a portrait at home during their lifetimes which would later be used as mummy portrait, it is hard to believe that sub-adults had those portraits at hand, since the individual facial features generally evolve rapidly during infancy and adolescence, so that those portraits would have needed to be renewed and adapted every few years. At the moment at least, there is no evidence that such a “monitoring” of a person’s appearance was common for the Graeco-Roman period of Ancient Egypt. This in turn strongly suggests that the mummy portraits were made post-mortem–which might be in line with the observations of some portraits that contain “sketches” of the face on their dorsal side [1].

Until now, there have been no comparative studies investigating the concordance–or divergence–between mummy portrait and individual facial traits in an infant mummy. In our study we found high concordance between both–as evidenced by both subjective visual evaluation and biometric analysis–though the match is not 100%. Interestingly, the range of concordance is in the same range as that given for forensic facial reconstructed cases where Miranda et al. [19] confirmed a 63–73% concordance rate. Furthermore, this means that the portrait does, indeed, depict the infant. In consequence, the portrait must have been finished either during its lifetime or shortly after death, before the body was embalmed.

There are, however, certain distinct differences between portrait and face: on a subjective level, the portrait appears slightly “older”; on a biometric level, the width of the nose and the mouth are smaller in the portrait than in the face, which might explain the perceived difference in age.

Besides this observation of concordance/ divergence there exists a study that shows specific paleoneurological disorders in a series of Fayoum portraits. Therefore, Appenzeller et al. [20] screened 200 mummy portraits–including one fixed to the skull which was investigated by CT-scan, however, without facial reconstruction. In this series 6 faces with neuropathological diagnoses could be identified which ranged from facial hemiatrophy to deviation of visual axes and corectopia (oval deviation of the pupils). Although the portrait in our present study does not provide pathological features, the presence of particular disease in several other images is a further argument that the portraits have been furnished specifically for the deceased individual.

The CT-investigation provides further information on the infant beyond the facial reconstruction: besides the supplementary anthropological information on the individual’s age and sex, the examination showed the typical manipulations of ancient Egyptian embalming, including transsphenoidal removal of the brain, exenteration of the abdomen and bandaging. Additionally, the typical features of post-mortal shrinkage are detectable. However, at variance to the typical features of ancient Egyptian embalming, only the abdominal organs seem to have been removed, while the lungs remained in place.

The CT-scans provide additional evidence for pathological alterations that may even have led to the infant´s death. Although there exists no information about biographical data–a general problem of paleopathological research [21]–the observations provide insight into the underlying presumed disease and its obvious consequences in this particular historic individual. Despite the usual embalming tradition, the embalmer had not removed the thorax organs–the lungs and the heart–form the infant´s body. Accordingly, the CT-scans reveal the right lung with a diffuse thickening of the parenchyma, while the left lung is present in the left thoracic cavity typically shrunken and attached to the dorsal chest wall. The expanded and thickened lung is highly suggestive of an infiltration of the lung during inflammation, such as in pneumonia.

Although a final verification of an inflammatory pulmonary disease is impossible–the removal of tissue samples for histological and/or molecular verification of inflammation residues and/or specific microbial identification is in the very well preserved and completely enveloped mummy obsolete–the diagnosis of pneumonia is highly conceivable.

In the differential diagnosis for lung tissue enlargement pulmonary bleeding, proteinoses, surfactant disorders, pulmonary interstitial glycogenosis, and neuroendocrine cell hyperplasia of infancy (as well as other general metabolic disorders) are excluded by the unilateral type of lesion [22]. Similarly, inflammation of autoimmune diseases is very unlikely for the same reason [22]. On the other hand, the isolated affection of one lung lobe (or only part of a lobe) is observed in circumscribed tumours (e.g. chondrohamartoma, teratoma and others), lung sequestration, cysts, emphysematous bullae and localized inflammation (pneumonia) [23] The CT-scans exclude a circumscribed mass, such as in tumours, cysts or emphysematous bullae so that finally the differential diagnosis leaves lung sequestration and localized pneumonia as differential diagnosis.

Taking into account that lung sequestration is a very rare disorder and usually shows affection of only a small portion of the lung lobe [23], the pneumonic inflammatory infiltration is the most probable diagnosis. Pneumonia mostly is the result of aerogenic bacterial infection affecting a part of the lung (bronchopneumonia) or a complete lobe (lobar pneumonia) [24]. Etiologically, a non-specific bacterial infection has to be differentiated from a specific–mostly mycobacterial–infection (tuberculosis) [24].

In paleopathology, there exist only very limited observations on this issue–mostly due to the fact that the lungs usually have been removed during exenteration. However, recently we observed in an ancient Egyptian infant mummy adhesions between the lung and the chest wall as residues of a (molecularly proven) tuberculous infection of the lung [25]. Further data on infantile mummy are also lacking in larger compilations of data [26].

It is therefore conceivable that the infant suffered from a significant lung inflammation (broncho- or lobar pneumonia) which is also very likely regarded as the cause of death, since usual bacterial pneumonia frequently leads to death in historic as well recent populations or different age levels. Finally, there is no evidence that the deformity of the thoracic cage (*pectus carinatum*) may have promoted the development of any pneumonia since the deformity usually does not lead to functional restrictions [27].

In summary, although we have no further information on the deceased’s identity, we were able to find out more about its physical state: the 3–4 years old boy probably suffered from severe pneumonia that may have led to his premature death. The body was embalmed according to the typical ritual of the Graeco-Roman period, including the painting and application of a mummy portrait. The comparison between portrait and facial reconstruction strongly suggests that the portrait represented the deceased as he really looked. It must therefore have been made shortly after his death–possibly with the help of preliminary sketches. The portrait itself presumably followed certain artistic specifications or norms that may have resulted in the subject’s face looking older than his real age.

Since we are only able to provide a single case, it remains to be determined whether it is unique or part of a general phenomenon. Further studies will hopefully resolve this question.

## Supporting information

S1 Video Recon-1. (MP4)

S2 Video Recon-2. (MP4)

S3 Video Recon-3. (MP4)

S4 Video Recon-4. (MP4)
